# Fat Oxidation, Hormonal and Plasma Metabolite Kinetics during a Submaximal Incremental Test in Lean and Obese Adults

**DOI:** 10.1371/journal.pone.0088707

**Published:** 2014-02-11

**Authors:** Stefano Lanzi, Franco Codecasa, Mauro Cornacchia, Sabrina Maestrini, Alberto Salvadori, Amelia Brunani, Davide Malatesta

**Affiliations:** 1 Institute of Sport Sciences University of Lausanne (ISSUL), University of Lausanne, Lausanne, Switzerland; 2 Department of Physiology, Faculty of Biology and Medicine, University of Lausanne, Lausanne, Switzerland; 3 Pulmonary rehabilitation department, San Giuseppe Hospital, *Istituto Auxologico Italiano* Piancavallo, Verbania, Italy; 4 Molecolar biology laboratory, San Giuseppe Hospital, *Istituto Auxologico Italiano* Piancavallo, Verbania, Italy; 5 Medicine rehabilitation department, San Giuseppe Hospital, *Istituto Auxologico Italiano* Piancavallo, Verbania, Italy; INSERM/UMR 1048, France

## Abstract

This study aimed to compare fat oxidation, hormonal and plasma metabolite kinetics during exercise in lean (L) and obese (O) men. Sixteen L and 16 O men [Body Mass Index (BMI): 22.9±0.3 and 39.0±1.4 kg^.^m^−2^] performed a submaximal incremental test (Incr) on a cycle-ergometer. Fat oxidation rates (FORs) were determined using indirect calorimetry. A sinusoidal model, including 3 independent variables (dilatation, symmetry, translation), was used to describe fat oxidation kinetics and determine the intensity (Fat*_max_*) eliciting maximal fat oxidation. Blood samples were drawn for the hormonal and plasma metabolite determination at each step of Incr. FORs (mg^.^FFM^−1.^min^−1^) were significantly higher from 20 to 30% of peak oxygen uptake (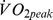) in O than in L and from 65 to 85% 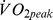 in L than in O (*p≤*0.05). FORs were similar in O and in L from 35 to 60% 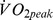. Fat*_max_* was 17% significantly lower in O than in L (*p*<0.01). Fat oxidation kinetics were characterized by similar translation, significantly lower dilatation and left-shift symmetry in O compared with L (*p*<0.05). During whole exercise, a blunted lipolysis was found in O [lower glycerol/fat mass (FM) in O than in L (*p*≤0.001)], likely associated with higher insulin concentrations in O than in L (*p*<0.01). Non-esterified fatty acids (NEFA) were significantly higher in O compared with L (*p*<0.05). Despite the blunted lipolysis, O presented higher NEFA availability, likely due to larger amounts of FM. Therefore, a lower Fat*_max_*, a left-shifted and less dilated curve and a lower reliance on fat oxidation at high exercise intensities suggest that the difference in the fat oxidation kinetics is likely linked to impaired muscular capacity to oxidize NEFA in O. These results may have important implications for the appropriate exercise intensity prescription in training programs designed to optimize fat oxidation in O.

## Introduction

Obesity is associated with a variety of health-related risks, such as hypertension and type 2 diabetes, all of which may center around insulin resistance [1]. Moreover, insulin resistance seems to be linked with an impaired ability to oxidize lipids in the skeletal muscle in obesity [2]. Endurance exercise training at intensity (Fat*_max_*) eliciting maximal fat oxidation (MFO) may enhance fat oxidation, muscle oxidative capacity [3] and insulin sensitivity [4] in obese individuals, suggesting its pivotal role in weight management in this population.

There is equivocal evidence concerning the effect of obesity on the fat oxidation rates (FORs) during exercise [5]–[10]. MFO and Fat*_max_* were found to be lower in obese compared with lean individuals [5]. However, recently, Ara et al. [10] suggest that MFO and Fat*_max_* were higher in class I obese individuals than in lean controls when groups were matched for aerobic fitness [similar maximal oxygen uptake (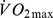)]. In contrast, when these groups were not matched for aerobic fitness, no difference were found in MFO, Fat*_max_* or FORs during low and moderate exercise intensities (∼35–70% 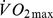 [11]) [7]. To date, little is known about MFO, Fat*_max_* and FORs over a large range of exercise intensities and especially during high intensity exercise (>64% 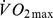 [11]) in obese individuals with a high body mass index (BMI). In fact, it is realistic to think that such individuals may not be matched with regard to aerobic fitness with lean control counterparts. Consequently, it is supposed that FORs over a large range of exercise intensities and Fat*_max_* may be at least similar to or lower in obese individuals with a high BMI than in lean individuals. Moreover, previous studies [7], [10] principally focused on the muscular factor with less emphasis on the extra-muscular factors (hormones and plasma metabolites) that regulate fat metabolism during exercise. In fact, the latter may be altered by differences in substrate availabilities and lipolytic hormones between lean and obese individuals, especially at high exercise intensities [12]–[15]. This may induce a narrowing of the whole-body fat oxidation kinetics and a lower Fat*_max_* zone (i.e., the range of exercise intensities with fat oxidation rates within 10% of MFO [16]). For a clinical standpoint, this implies that the ‘individualization concept of training’ must be taken into account for weight management training programs, especially in metabolic disease [17].

This study aimed to quantitatively characterize and compare whole-body fat oxidation, hormonal and plasma metabolite kinetics over a large range of intensities during a submaximal incremental test in obese with high BMI and lean adults. It was hypothesized that differences in non-esterified fatty acid (NEFA) availability and hormonal milieu between the two groups may decrease FORs at high exercise intensities, inducing a narrowing of the whole-body fat oxidation kinetics in obese compared with lean individuals. However, Fat*_max_*, MFO and FORs at low and moderate intensities may be similar between groups.

## Methods and Procedures

### Subjects

Sixteen young sedentary lean (L) and 16 young obese (class I: n = 4, class II: n = 7, and class III: n = 5) men (O) were recruited to participate in this study (Table 1). O were patients recruited from the *Istituto Auxologico Italiano* (Piancavallo, Italy) where they spent 4 weeks. During the first week subjects had physical examination and clinical routine analysis. Thereafter, they followed a 3-week personalized lifestyle education program including dietary [a balanced diet individually prescribed by the nutritionist of the institute (57±1% CHO, 25±0% fat, 18±1% protein, 2045±49 Kcal^.^d^−1^)], recreational activities at self-controlled intensity and psychological follow-up. The testing session were conducted at the end the hospitalization program (∼29 days) when the weight fluctuations were minimal (see methodological discussion). L individuals were recruited and followed a 4-day balanced diet [with the same macronutrient proportion and with an energy intake corresponding to ∼2000 Kcal^.^d^−1^] preceding the testing session. Before the beginning of the testing session, all the subjects have confirmed that they followed the nutritional indications. Subjects with hypertension [blood pressure >130/90 mmHg], impaired fasting glucose (>6.1 mmol^.^L^−1^) [18], type 2 diabetes and abnormal electrocardiogram at rest were excluded. None of the subjects were using any medication known to influence energy metabolism. Insulin sensitivity was assessed by homeostasis assessment of insulin resistance (HOMA-IR) [19]. The study was approved by the Ethics Review Committee of the *Istituto Auxologico Italiano*, Italy. All subjects provided written, voluntary, informed consent before participation. The experiment was conducted according to the Declaration of Helsinki.

**Table 1:** Characteristics of the study subjects.

	Lean	Obese	P value
N	16	16	-
Age, yr	33.1±1.6	34.5±2.1	NS
Weight, kg	72.4±1.2	121.3±4.8	≤ 0.001
Height, m	1.78±0.01	1.76±0.02	NS
BMI, kg^.^m^−2^	22.9±0.3	39.0±1.4	≤ 0.001
Fat mass, kg	13.7±1.0	51.8±3.8	≤ 0.001
Fat mass, %	19.8±1.3	42.4±1.4	≤ 0.001
Fat free mass, kg	55.7±1.3	68.1±1.7	≤ 0.001
Fasting glucose, mmol^.^L^−1^	5.2±0.1	5.3±0.1	NS
Fasting insulin, mU^.^L^−1^	4.5±0.9	15.9±4.1	< 0.01
HOMA-IR	1.0±0.2	3.8±1.0	≤ 0.001

Values are the means±SE. BMI: body mass index; HOMA-IR: homeostasis assessment of insulin resistance; NS: non significant.

### Preliminary testing

All subjects underwent dual-energy X-ray absorptiometry (DEXA) for measurements of body composition (DPX-IQ X-ray bone densitometer version 4.7e, USA). For O whose body weight exceeded 125 kg (n = 6), body composition was assessed using a tetrapolar bioelectrical impedance method (BIA 101/S, Italy) [20], and the hydration of each subject was controlled with bioimpedance vector analysis [21]. As BIA tended to overestimate the fat free mass (FFM) in O [22], for these 6 subjects, we applied a linear regression between DEXA and BIA of a sample of 20 overweight/obese hospitalized men patients (BMI range: 25 – 52 kg^.^m^−2^) to transform the FFM BIA’s data. Repeated regressions removing one subject at a time allowed us to assess the accuracy of the linear regression. Peak oxygen uptake (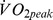) and peak power output (PPO) were determined by a maximal ramp incremental test to exhaustion on a cycle-ergometer (Ebike Basic BPlus, USA). After a 3-min rest period, O started with a 5-min warm-up at 40 W (60 W for L), after which the PO was linearly increased by 20 W (30 W for L) every minute until exhaustion. 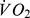, carbon dioxide production (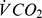) and ventilation (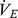) were measured continuously using a breath-by-breath online system (V*_max_* 229, Sensor Medics, USA). Heart rate (HR) was recorded continuously using an HR monitor (Polar RS800, Finland). 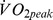 was defined as the highest 10-s mean value recorded before the subject’s volitional termination of the test, whereas PPO was defined as the highest peak value reached during the maximal incremental ramp test.

### Experimental protocol

Seven days after the preliminary test, the experimental trial was performed in the morning (between 0800-0900 hours) after a minimum 12-h overnight fast. All participants were asked to refrain from exercise, alcohol, and caffeine for the 24-h period preceding the test. After a 15-min seated resting period, the subjects remained seated for 15 min on the cycle-ergometer and were connected to the metabolic system (Rest). Average HR and gas exchange data during the final 2 min were used as the baseline. Thereafter, subjects performed a submaximal incremental test (Incr) to determine the whole-body fat oxidation kinetics. After a standardized 10-min warm-up at 20% PPO, the PO was increased by 7.5% PPO every 6 min until 65% PPO or until the respiratory exchange ratio (RER) reached 1.0. HR and respiratory values were averaged over the last minute of each stage.

The blood samples were drawn at Rest and during the last 3 min of the warm-up and each step to determine serum insulin and NEFA, plasma epinephrine (E), norepinephrine (NE), glycerol, atrial natriuretic peptide (ANP), glucose and lactate concentrations. The blood samples were collected through an indwelling cannula inserted at the antecubital vein, which was kept patent by continuous slow saline infusion. All blood aliquots were placed on ice, and after the final step were centrifuged at 4°C and 3,000 G for 10 min. Plasma or serum samples were transferred to storage tubes and frozen at –80°C until analysis.

### Data analysis and calculations

*Indirect calorimetry.* Fat and CHO oxidation rates were calculated using stoichiometric equations [23]. The results of the Incr were used to calculate FORs over a wide range of exercise intensities. To model whole-body fat oxidation kinetics, represented as a function of exercise intensity, and determine Fat*_max_* and MFO, the SIN model was used [24]. The SIN model includes 3 independent variables, representing the main quantitative characteristics of the curve: dilatation, symmetry, and translation (Equation 1).
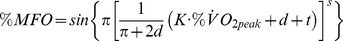
(1)where *d*, *s*, and *t* are the dilatation, symmetry, and translation variables, respectively, and *K* is the constant of intensity, which corresponds to (π /100). Dilatation refers to the degree of dilatation or retraction of the curve; the symmetry variable is used to break the symmetry of the standard basic sine curve, and translation refers to the translation of the whole curve toward the abscissa axis [24]. The FORs were determined at intervals of 5% 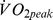 between 20–85% 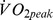. The Fat*_max_* zone was determined by calculating the range of exercise intensities with fat oxidation rates within 10% MFO [16]. In addition, %HR*_max_* and RER (RERFat*_max_*) at Fat*_max_* were determined. Delta efficiency (DE) was calculated as previously described [25].

*Blood samples.* Serum insulin concentrations were measured by chemiluminescence (Immulite 2000 Analyzer, USA). The insulin assay sensitivity was 2 µlU^.^mL^−1^; the inter- and intra-assay coefficients of variation (CVs) were 4.0 and 5.1%, respectively. Serum NEFA concentrations were analyzed spectrophotometrically using commercial enzymatic and colorimetric kits (Randox Laboratories, USA). The NEFA assay sensitivity was 0.072 mmol^.^L^−1^; the inter- and intra-assay CVs were 4.51 and 4.74%, respectively. The E and NE plasma concentrations were determined with high-performance liquid chromatography (HPLC) using kits from Chromosystems (Chromsystems Instruments & Chemicals GmbH, Germany). The sensitivities of these assays and the inter- and intra-assay CVs were as follows: 10 ng^.^L^−1^, 4.0 and 2.9% for E, and 15 ng^.^L^−1^, 1.7 and 3.7% for NE, respectively. Plasma glycerol concentrations were measured using an enzymatic and colorimetric commercial assay kit (Cayman Chemical Company, USA); the inter- and intra-assay CVs were 6.5 and 6.5%, respectively. Samples for plasma ANP were preconditioned with aprotinin (500 Kallikrein inhibition units per mL), and plasma ANP concentrations were determined using a EURIA kit with prior extraction (EURO-Diagnostic, Sweden; sensitivity 3.5 pg^.^mL^−1^); inter- and intra-assay CVs were 11.6% and 8.6%, respectively. Plasma glucose and lactate concentrations were assessed using an amperometric method (Gem Premier 3000; Instrumentation Laboratory, USA).

### Statistical analysis

A 2-way repeated-measures mixed design ANOVA followed by contrasts was performed to compare RER and fat oxidation at absolute PO [exercise intensity (n = 9; 30–150W) x group (obese *vs.* lean)] and RER, HR, 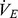, and fat oxidation at each relative exercise intensity (%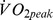) [exercise intensity (n = 14; 20–85% 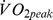) x group (obese *vs.* lean)]. This test was also used to compare plasma metabolite and hormonal kinetics at rest and at each relative exercise intensity (% PPO) [exercise intensity (n = 7; Rest-57.5% PPO) x group (obese *vs.* lean)] during Incr between L and O. A *t* test (or Mann–Whitney rank sum test for nonparametric values) was used to identify differences in the parameters (Fat*_max_*, Fat*_max_* zone and MFO) and in the SIN model variables (dilatation, symmetry and translation) of the whole-body fat oxidation kinetics obtained during Incr. These tests were also used to determine differences in anthropometric and physical characteristics between O and L. Significance was set at *p*≤0.05.

## Results

### Anthropometric characteristics and maximal incremental ramp test

There was no significant difference in age and height between the two groups (Table 1). Weight, BMI, fat mass (FM) and FFM were significantly higher in O compared with L (Table 1). Fasting glucose was similar in O and L, whereas fasting insulin and HOMA-IR were significantly higher in O compared with L (Table 1). 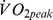 (mL^.^min^−1.^kg^−1^ and mL^.^FFM^−1.^min^−1^), PPO, and HR*_max_* were significantly lower in O than in L, whereas 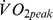 (mL^.^min^−1^) was similar in the two groups (Table 2).

**Table 2:** Maximal incremental ramp test and characteristics of whole-body fat oxidation kinetics during the submaximal incremental test (Incr).

	Lean	Obese	P value
**Maximal incremental ramp test**			
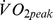 , mL^.^min^−1^	3003±138	3052±106	NS
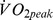 , mL^.^min^−1.^kg^−1^	41.8±1.8	25.2±0.9	≤ 0.001
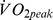 , mL^.^FFM^−1.^min^−1^	53.8±1.7	44.9±1.3	≤ 0.001
PPO, W	259±13	213±7	< 0.01
HR_max_, bpm	183±2	168±2	≤ 0.001
**Submaximal incremental test**			
MFO, mg^.^FFM^−1.^min^−1^	6.3±0.4	6.1±0.3	NS
Fat_max_, % 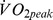	56.8±1.7	47.2±2.6	< 0.01
Fat_max_zone, % 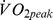	29.4±1.0	25.1±1.3	< 0.05
Fat_max_, % HR_max_	73.8±1.7	65.2±2.3	< 0.01
RERFat_max_	0.88±0.00	0.83±0.01	≤ 0.001
Dilatation	0.2±0.1	–0.1±0.1	≤ 0.001
Symmetry	1.2±0.1	1.0±0.0	< 0.05
Translation	0.0±0.1	0.1±0.1	NS

Values are the means±SE. 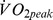: peak oxygen uptake; FFM: fat-free mass; PPO: peak power output; HR*_max_*: maximal heart rate; Fat*_max_*: exercise intensity at which maximal fat oxidation rate (MFO) occurs; Fat*_max_* zone: range of exercise intensities with fat oxidation rates within 10% MFO; RER: respiratory exchange ratio; NS: non significant.

### Pre-exercise resting period


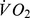
 (6.1±0.3 and 6.3±0.3 mL^.^FFM^−1.^min^−1^), HR (73±2 and 74±3 beats^.^min^−1^), 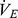 (10.7±0.4 and 11.0±0.7 L^.^min^−1^) and FORs (2.5±0.3 and 1.9±0.2 mg^.^FFM^−1.^min^−1^) were similar in O and in L, respectively. RER was significantly lower in O than in L (0.76±0.01 and 0.83±0.02, respectively; *p*≤0.01).

### Submaximal incremental test

**Figure 1:**
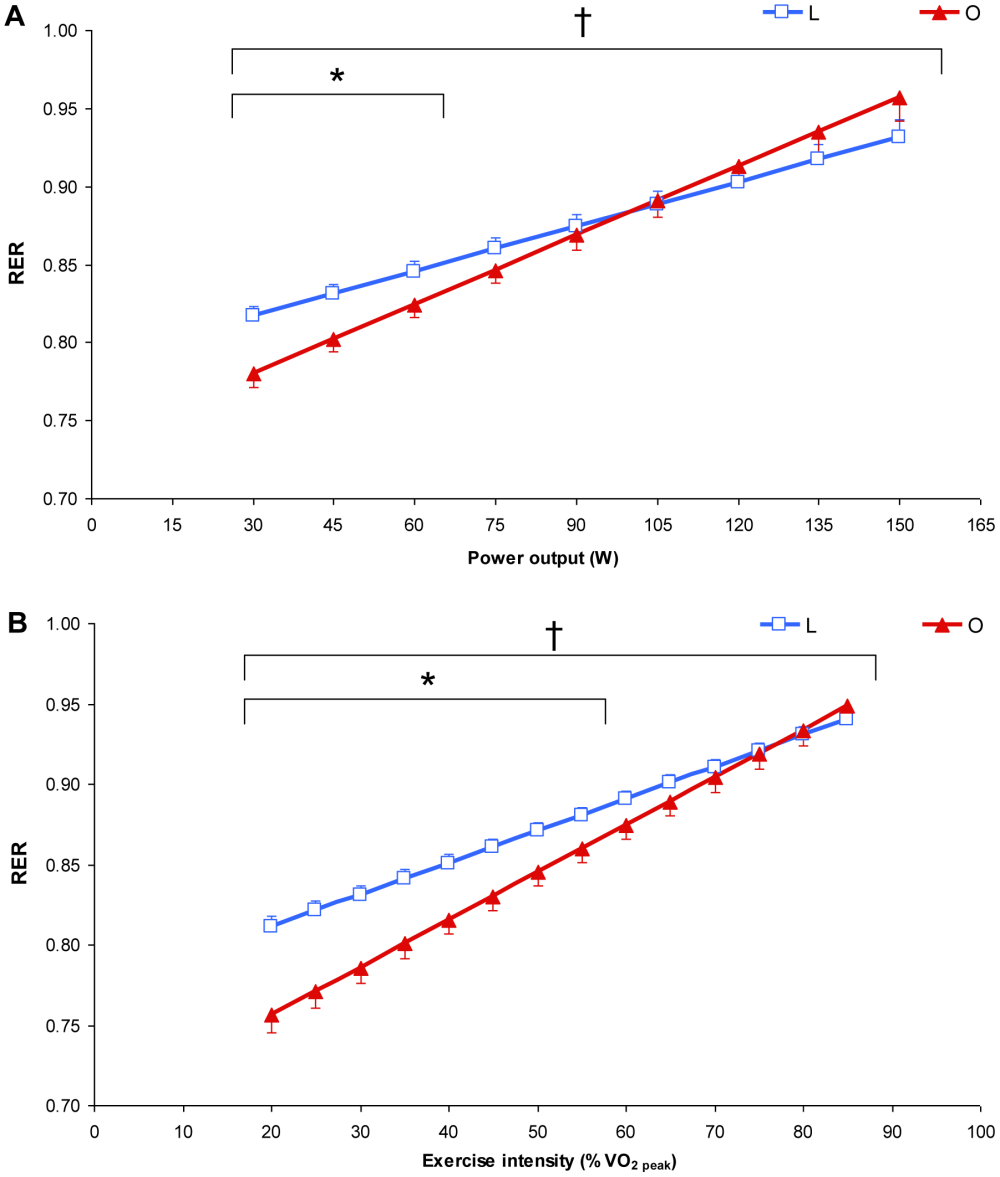
Mean respiratory exchange ratio (RER) values represented as a function of exercise intensity [absolute power output (A) and % of peak oxygen uptake (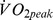) (B)] determined during the submaximal incremental test in lean (L: blue, n = 16) and obese (O: red, n = 16) individuals. Values are the means±SE. * *p≤*0.05 for differences with lean; † *p≤*0.05 for significant group interaction effect.

**Figure 2:**
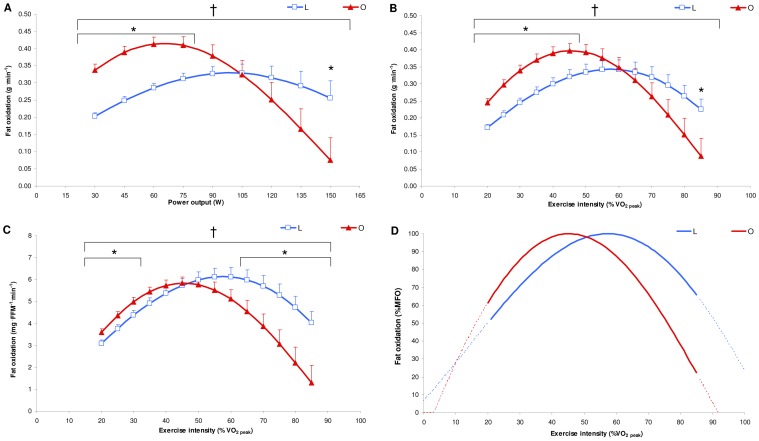
Mean whole-body fat oxidation kinetics in absolute [g^.^min^−1^ (A and B) and mg^.^FFM^−1.^min^−1^ (C)] and relative [% of maximal fat oxidation (MFO) (D)] values determined with the sinusoidal (SIN) model and during the submaximal incremental test in lean (L: blue, n = 16) and obese (O: red, n = 16) individuals. Values are the means±SE. 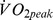: peak oxygen uptake. * *p≤*0.05 for differences with lean; † *p≤*0.05 for significant group interaction effect.

RER showed a significant interaction effect (*p≤*0.01) and was significantly lower from 30 to 60 W in O than in L (Figure 1A, *p<*0.05) and from 20 to 55% 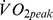 in O than in L (Figure 1B, *p<*0.05). FORs, expressed in g^.^min^−1^, showed a significant interaction effect (*p*≤0.001) and were significantly higher from 30 to 75 W in O than in L (*p*≤0.01) and at 150 W in L than in O (Figure 2A, *p*<0.05). FORs, expressed in g^.^min^−1^, showed a significant interaction effect (*p*≤0.001) and were significantly higher from 20 to 45% 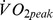 in O than in L (*p*≤0.01) and at 85% 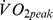 in L than in O (Figure 2B, *p*<0.05). FORs, expressed in mg^.^FFM^−1.^min^−1^, showed a significant interaction effect (*p≤*0.001) and were significantly higher from 20 to 30% 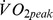 in O than in L (*p≤*0.05) and from 65 to 85% 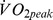 in L than in O (Figure 2C, *p≤*0.05). Whole-body fat oxidation kinetics were characterized by similar translation, significantly lower dilatation and left-shift symmetry in O compared with L (Table 2, Figure 2D). MFO was similar in O and in L, and Fat*_max_*, Fat*_max_* zone and RERFat*_max_* were significantly lower in O compared with L (Table 2). HR showed a significant interaction effect (*p≤*0.001) and was significantly lower from 35 to 85% 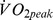 in O than in L (data not shown, *p<*0.05). DE was similar in O and L (18.6±0.5 and 19.8±0.7%, respectively). 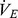 and 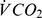 respiratory equivalent showed no significant main group effect and no significant interaction effect (data not shown).

### Plasma metabolite and hormonal concentrations

**Figure 3:**
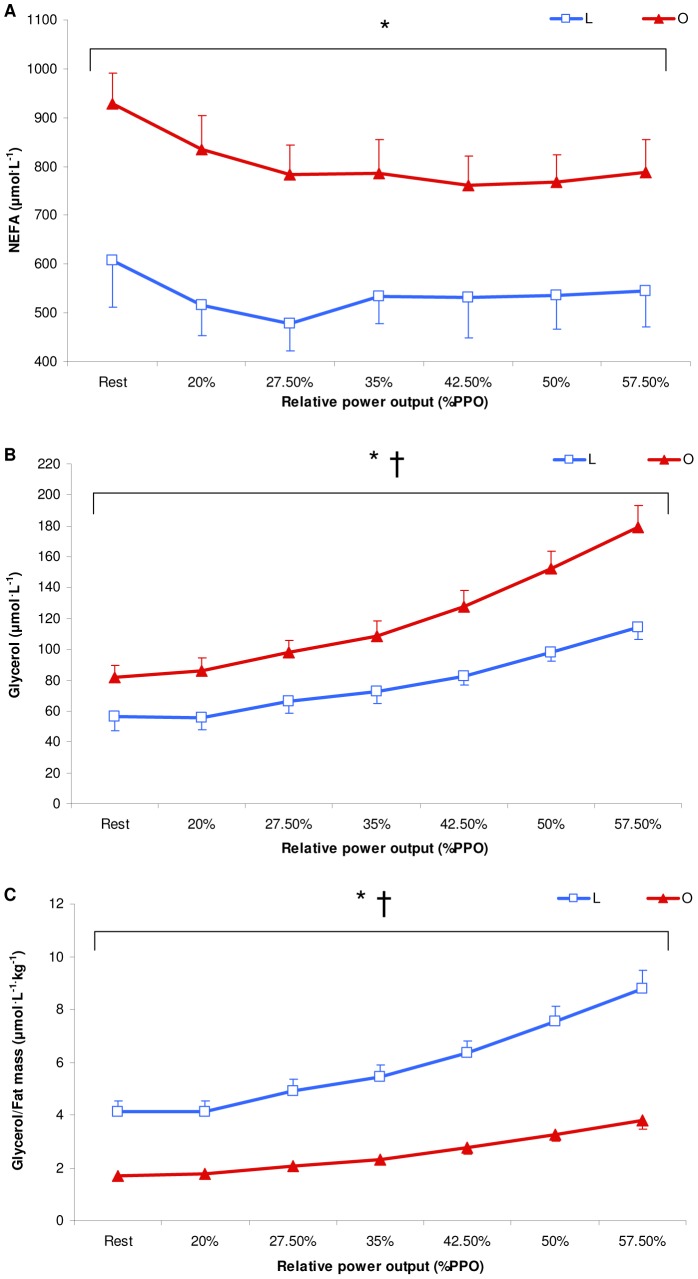
Mean non-esterified fatty acid (NEFA) concentrations (A) and mean glycerol concentrations (B) divided by fat mass (C) during the submaximal incremental test in lean (L: blue, n = 16) and obese (O: red, n = 14) individuals. Values are the means±SE. PPO: peak power output. * *p≤*0.05 for differences with lean; † *p≤*0.05 for significant group interaction effect.

There were no significant main group and interaction effects for plasma glucose and lactate concentrations (data not shown). Plasma NEFA concentrations showed a significant main group effect (*p<*0.01) with no significant interaction effect and were significantly higher at Rest and for all exercise intensities in O than in L (Figure 3A, *p*<0.05). Plasma glycerol concentrations showed a significant interaction effect (*p≤*0.001) and were significantly higher at Rest and for all exercise intensities in O than in L (Figure 3B, *p*<0.05). Plasma glycerol concentrations divided by kg of FM were significantly lower at Rest and for all exercise intensities in O than in L [Figure 3C, *p*≤0.001; significant interaction effect (*p≤*0.001)].

**Figure 4:**
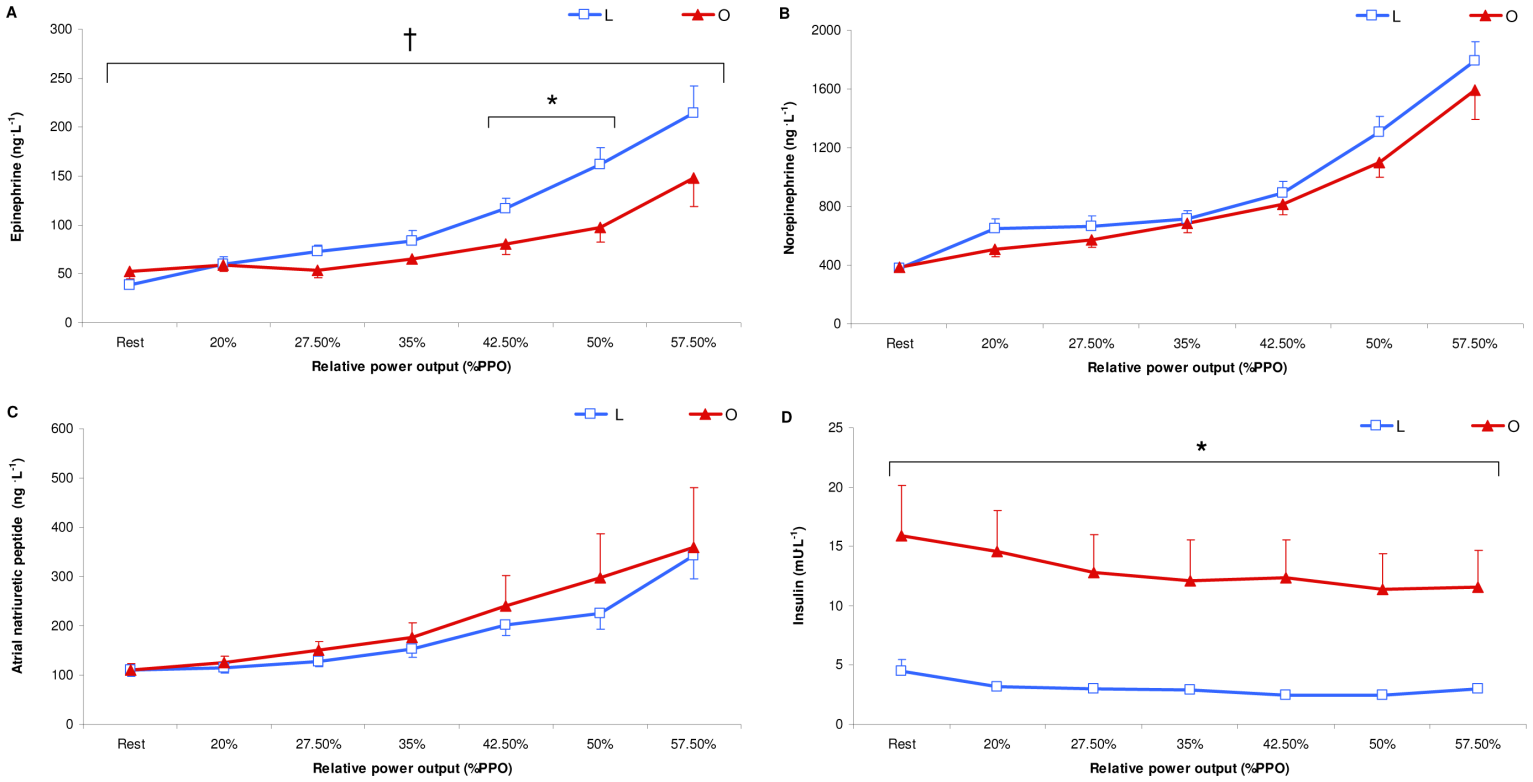
Mean epinephrine (A), norepinephrine (B), atrial natriuretic peptide (C) and insulin (D) concentrations during the submaximal incremental test in lean (L: blue, n = 16) and obese (O: red, n = 14) individuals. Values are the means±SE. PPO: peak power output. * *p≤*0.05 for differences with lean; † *p≤*0.05 for significant group interaction effect.

Plasma E concentrations showed a significant interaction effect (*p<*0.01) and were significantly lower at 42.5 and 50% PPO in O than in L (Figure 4A, *p<*0.05). There were no significant main group or interaction effects for plasma NE and ANP concentrations (Figures 4B and 4C, respectively). Plasma insulin concentrations showed a significant main group effect (*p<*0.01), with no significant interaction effect, and were significantly higher at Rest and for all exercise intensities in O than in L (Figure 4D, *p*<0.01).

## Discussion

The results of this study showed that O presented a lower Fat*_max_*, a left-shifted and less dilated curve and a lower reliance on fat oxidation at high but not at low or moderate exercise intensities. Moreover, MFO was similar in the two groups. Lipolysis (attested by glycerol/FM concentrations) was found to be lower in O during all exercise intensities, and blunted lipolysis was most likely associated with lower E concentrations (significant interaction effect between the two groups) and/or hyperinsulinemia. Despite the blunted lipolysis, O presented higher NEFA availability, most likely due to larger amounts of FM. Therefore, contrary to our hypothesis, these results suggest that the decreased FORs in O relative to L at high exercise intensities are not associated with decreased plasma NEFA availability, but most likely linked to impaired muscular capacity to oxidize NEFA.

The absolute 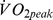
values were similar between O and L counterparts and are in line with previous studies that tested, on cycle-ergometer, individuals with similar class of obesity [6], [8], [26], [27]. However, as our O presented lower HR*_max_* values compared to L individuals during the maximal ramp incremental test, 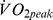 may be likely underestimated in O individuals and representing variables as a function of %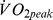 would interfere with the comparison of the two groups. For this reason, before to represent our results according to the %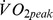, RER and FORs have been expressed and analyzed as a function of absolute PO, which was actually measured during Incr. However, this analysis gives similar results compared with that expressing these variables as a function of %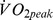 (Figures 1 and 2). Moreover, it has been suggested that 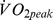 may also be indicative of a true maximal oxygen consumption (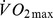) in lean [28] and in obese individuals [29] and previous studies compared FORs in obese and lean individuals reporting FORs as a function of 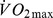 [7], [10]. Therefore, representing variables as a function of %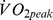 may be a reasonable choice for both groups and may add important information of difference in FORs in L and O individuals. Moreover, this allows us to compare our results to previous findings [7], [10].

Our results, according to previous findings [6], [30], showed that RER was lower in obese compared to lean counterparts, indicating a greater proportion of energy derived from fat during low-to-moderate exercise intensities. Indeed, the fat oxidation kinetics, expressed in g^.^min^−1^, present higher values in O than in L during low intensities (i.e., 20–45% 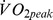, Figure 2B), confirming those of Ara et al. [10], who showed higher rates of fat oxidation between groups matched for aerobic fitness and with similar FFM. Contrary to Ara et al. [10], our results showed that obese individuals presented lower FORs during high exercise intensities inducing therefore a significant interaction effect between the two groups. However, as our O presented higher FFM values, these observations report only indications of the global fat oxidation irrespective of differences in FFM between the two groups. Therefore, expressing the FORs in mg^.^FFM^−1.^min^−1^ seems to be more judicious with regard to obtaining information about fat oxidation at the skeletal muscle level. Our results showed that the FORs per kg of FFM were higher in O than in L during low exercise intensities (20–30% 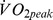), similar during moderate exercise intensities (50–60% 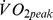) and higher in L than in O during high exercise intensities (65–85% 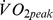; Figure 2C). Moreover, the significant interaction effect clearly demonstrates that the pattern of fat oxidation kinetics was different in the two groups, substantially confirming the findings of FORs expressed in g^.^min^−1^. In addition, as the FORs may be affected by the aerobic fitness [31], the SIN model, using relative FORs values (%MFO, Figure 2D), allows us to compare the fat oxidation kinetics independently of differences in aerobic fitness between the two groups. This comparison showed that obese individuals presented a left-shifted and less dilated curve compared to lean individuals, associated with a lower reliance on fat oxidation at high exercise intensities. The different profiles observed according to exercise intensity may be mainly attributable to differences between the two groups in 1) the modulation of lipolysis in adipose tissue (AT) and NEFA availability for skeletal muscle and/or 2) the lipid oxidation in skeletal muscle during exercise.

Our findings showed that glycerol/FM concentrations were lower in O than in L for all exercise intensities with a significant interaction effect between groups, suggesting blunted lipolysis in O. This finding may be explained by the lower E concentrations at moderate intensities in O than in L and by the significant interaction effect for E between L and O (Figure 4A). In fact, it has been recently shown that E, and not NE, is a determinant of exercise-induced lipid mobilization in human subcutaneous AT [32]. Moreover, as E concentrations were not significantly different at rest and during low exercise intensities between the two groups, higher insulin concentrations may also contribute to the blunted lipolysis in O [8]. In addition, although previous studies suggested that the plasma ANP (a stimulator of lipolysis in AT [33]) is lower in obese than in lean individuals [34] (most likely linked to a higher expression of natriuretic peptide clearance receptors in AT with obesity [35]), our results showed no difference between O and L at Rest and during exercise. Indeed, it has been suggested that ANP receptor expression in AT was reduced in obese hypertensive but not in non-hypertensive obese individuals [35], suggesting that the alteration of ANP-induced cardio-metabolic actions may be related to hypertension [36]. However, because our O were not hypertensive, it is difficult to relate the blunted lipolysis in O with lower ANP receptor expression in AT.

Interestingly, higher absolute concentrations of NEFA and glycerol were found during all exercise intensities in O, suggesting that higher NEFA availability may be due to larger amounts of FM in these individuals [37]. Moreover, the NEFA concentration profiles display similar kinetics in both groups, indicating that NEFA availability cannot explain the different patterns of fat oxidation kinetics with regard to exercise intensities between the 2 groups. Therefore, contrary to our hypothesis, the lower FORs in O relative to L at high exercise intensities were not due to decreased plasma NEFA availability. Furthermore, despite a continuous increase in lipolysis (Figure 3), the stable plasma NEFA concentrations observed during Incr may suggest an enhanced NEFA uptake with respect to exercise intensities by skeletal muscle cells in both groups. Moreover, it has been suggested that extremely obese individuals (BMI: ∼ 40 kg^.^m^−2^) present a lower percentage of NEFA uptake oxidized during exercise compared to lean individuals [9], suggesting that NEFA that were taken up but not oxidized were re-esterified in the muscle, leading to enhanced rates of fat storage [6]. The reduced FORs at high exercise intensity may be linked to the decreased activity of the muscle carnitine palmitoyltransferase (CPT-1) and muscle citrate synthase (CS) (an index of mitochondrial content) in obese subjects relative to lean controls [1], [38], [39]. Although we do not know whether O presented higher NEFA uptake and/or decreased muscle CPT-1 and CS activity than L, we suggest that O may oxidize a lower percentage of NEFA uptake, leading to decreased FORs as the exercise intensity increases.

Moreover, as previously reported [8], [9], O can maintain total lipid oxidation at rest and during moderate exercise only if they can compensate for the reduction in plasma NEFA oxidation with enhanced intra-muscular triglyceride (IMTG) oxidation. The higher and similar FORs in O compared with L during low and moderate exercise intensities, respectively, seem to confirm this mechanism. Moreover, the latter results are in line with previous findings showing that similar insulin resistant obese individuals with normoglycemia used more fat and may have been more reliant to IMTG during low intensity exercise (∼45% 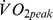) compared with insulin sensitive obese counterparts [40]. However, our results may indicate that this IMTG compensation may not be possible at high exercise intensities, leading to decreased FORs in O compared with L. In fact, it has been shown that IMTG oxidation decreases to a greater extent than plasma NEFA oxidation as the exercise intensity increases from moderate to high [41]. Thus, plasma NEFA oxidation, which represents a more important part of the total lipid oxidation during high exercise intensities, may exert a more substantial limiting effect and thus decrease the total lipid oxidation at these intensities in O. Therefore, we suggest that O may have a muscular defect in the ability to oxidize lipids [38], [39] at high exercise intensities, most likely due to decreased plasma NEFA oxidation [6], [9].

**Figure 5:**
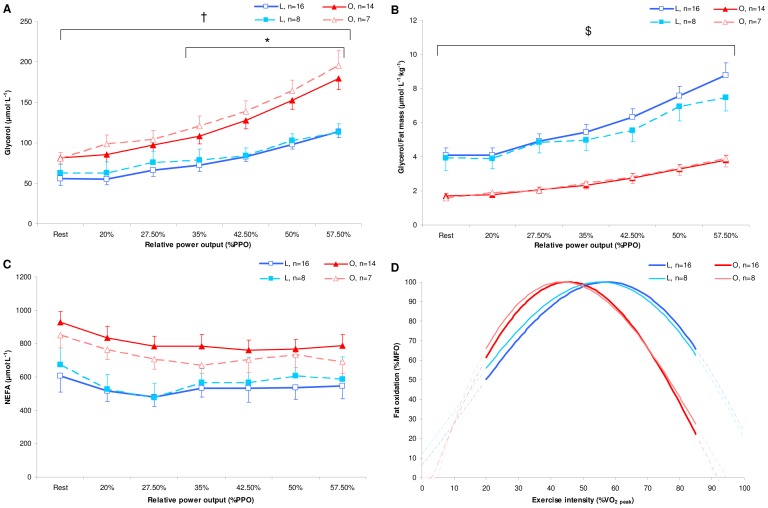
Mean glycerol concentrations (A) divided by fat mass (B), mean non-esterified fatty acid (NEFA) concentrations (C) and whole-body fat oxidation kinetics in relative (D) [% of maximal fat oxidation (MFO)] values determined with the sinusoidal (SIN) model during the submaximal incremental test in lean (L: dark and light blue) and obese (O: dark and light red) individuals. In the sub-groups matched for aerobic fitness, O present similar MFO (O: 6.3±0.6; L: 5.5±0.4 mg^.^FFM^−1.^min^−1^), left-shifted (O: 0.9±0.1; L: 1.2±0.1 for symmetry; *p*<0.05) and less dilated (O: –0.1±0.1; L: 0.3±0.1 for dilatation; *p*<0.05) curve, lower Fat*_max_* (O: 46.5±5.0; L: 55.8±2.6 %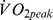) and lower Fat*_max_* zone (O: 25.8±2.3; L: 30.1±1.2 %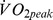) although non-significant as a consequence of the small sample size. Values are the means±SE. PPO: peak power output. * *p≤*0.05 for differences between sub-groups; † *p≤*0.05 for significant group interaction effect between sub-groups; $ for significant group effect between sub-groups [2-way repeated-measures mixed design ANOVA (exercise intensity x group) followed by contrasts].

Our results are in contrast with those of Ara et al. who found higher FORs during all exercise intensities and higher Fat*_max_* and MFO in obese compared with lean individuals. The reason for this discrepancy is unclear but may be a result of different factors, such as the degree of adiposity and the BMI (∼39 *versus* ∼34 kg^.^m^−2^ for O and ∼23 *versus* ∼27 kg^.^m^−2^ for L). Furthermore, the O in this study were not matched with regard to 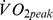/FFM; therefore, it is possible that the fat oxidation kinetics, MFO and Fat*_max_* results were influenced by differences in metabolic fitness and, thus, not directly associated with obesity. Therefore, we created a sub-group of 8 O (BMI: 39.1±2.0 kg^.^m^−2^) and 8 L (BMI: 23.5±0.4 kg^.^m^−2^) matched for 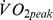/FFM (O: 48.7±1.6 mL^.^FFM^−1.^min^−1^; L: 48.8±1.7 mL^.^FFM^−1.^min^−1^, *p* = 0.94). This new comparison showed that the sub-group of obese individuals presented, as observed in the entire group (n = 16), a blunted lipolysis (Figure 5B) and higher NEFA availability (although non-significant as a consequence of the small sample size, Figure 5C) during all exercise intensities compared to lean individuals. In addition, despite this higher NEFA availability, the shape of fat oxidation kinetics during exercise was similar to that observed in the entire group (n = 16) and remains significantly left-shifted and less dilated compared to lean individuals (Figure 5D). This suggests that the lower reliance to fat oxidation at high exercise intensities may be directly associated with obesity and not with differences in aerobic fitness between the 2 groups.

Our results may also be relevant from a clinical standpoint and for exercise prescription in O. In fact, Fat*_max_* was found to be lower in O than in L, and its values were similar to those reported in the literature in lean [5], [31] and obese individuals [4], [7], [10]. The lower Fat*_max_* and Fat*_max_* zone in O compared to L suggest that the ‘individualization concept of training’ must be taken into account for weight management training programs. Training programs in class II and III O are rare, but targeting the training intensity in the zone that elicits MFO appears to be appropriate [4].

Some methodological limitations exist and need to be addressed. Firstly, our O were studied during a lifestyle education program, and therefore this condition may interfere with our findings and not be completely representative of the general obese population. However, the testing session was conducted at the end of the hospitalization program, when the weight changes were minimal. In addition, contrary to previous studies that performed only one day of diet control before the trial [5], [7], [10], our O followed a 3-week balanced diet before the testing session. Moreover, although our O may present a favourable condition to promote fat oxidation [42], [43], the decreased FORs observed during high exercise intensities and the lower dilatation may suggest that obese individuals really suffer from an impaired capacity to oxidize lipids. Secondly, although indirect calorimetry is extensively used to determine substrate oxidation during exercise, changes in the size of the bicarbonate pool may interfere with calculations of substrate oxidation at higher intensities [44]. However, it has been shown that close agreement exists between the estimates of substrate oxidation rates measured with indirect calorimetry and by an isotope method during strenuous exercise at ∼85% of 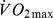 [45]. Moreover, our results of 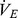 and 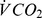 respiratory equivalent, represented as a function of exercise intensity, showed that there was no difference between groups, suggesting that indirect calorimetry may be accurately used to assess and compare substrate oxidation in the two groups.

In summary, this study showed that O with high BMI presented a left-shifted and less dilated curve and a lower reliance on fat oxidation at high but not at low or moderate exercise intensities. Despite the blunted lipolysis, O presented higher NEFA availability (most likely due to larger amounts of FM), suggesting that the decreased FORs in O at high exercise intensities are most likely linked to impaired muscular capacity to oxidize lipids. In addition, the different pattern of fat oxidation kinetics between the two groups may be directly associated with obesity and not with differences in aerobic fitness. The narrowing of the FORs and the lower Fat*_max_* and Fat*_max_* zone may have important implications for the appropriate exercise intensity prescription in training programs designed to optimize fat oxidation in O.
